# Neonatal Onset Seizures and Hypotonia Due to D-Bifunctional Protein Deficiency

**DOI:** 10.1007/s12098-025-05582-y

**Published:** 2025-05-22

**Authors:** Sohier Yahia, Dina Ghozzy, Yahya Wahba, Zahraa Abdelmoneim

**Affiliations:** Department of Pediatrics, Faculty of Medicine, Mansoura University, Mansoura, Egypt

**Keywords:** Peroxisomal disorder, Seizures, *HSD17B4*

## Abstract

Peroxisomal disorders (PDs) are a diverse group of inherited conditions arising from impaired function of a specific peroxisomal enzyme, metabolite transporter, or defect in the peroxisome biogenesis system. Peroxisomal D-bifunctional protein (DBP) deficiency is generally classified as a Zellweger-like syndrome. This disorder is caused by mutations in the *HSD17B4* gene, and only a limited number of confirmed cases have been reported to date.

The authors report case of a 6-mo-old female infant presenting with neonatal-onset intractable seizures, characteristic facial features, hypotonia, and progressive hepatomegaly. An acylcarnitine profile revealed elevated very long-chain fatty acids, prompting the initiation of a medium-chain triglyceride (MCT) formula. Remarkably, this treatment led to seizure control, improved muscle tone, and a reduction in liver size. Whole exome sequencing identified a homozygous missense mutation in the *HSD17B4* gene (c.1444A>T).

This case suggests that MCT-containing formulas may offer therapeutic potential in the treatment of D-bifunctional protein deficiency.

## Introduction

D-bifunctional protein (DBP) deficiency is an autosomal recessive disorder affecting peroxisomal fatty acid oxidation [1].

DBP deficiency symptoms typically begin in the neonatal period. Most infants present with hypotonia (98%) and seizures (93%) within the first month of life.

## Case Report

A 6-mo-old girl, born full-term to consanguineous parents (first cousins), presented with seizures starting at 3 wk of age. Her mother, gravida 3, parity 3, had a history of losing a son to neurodevelopmental delay and intractable seizures at 3 mo. The patient initially had tonic seizures, partially controlled with phenobarbital, but at 55 d, developed frequent uncontrolled tonic-clonic and focal seizures, unresponsive to maximum doses of levetiracetam, topiramate, and carbamazepine.

On examination, she appeared ill, with weight (3,200 g) and length (55 cm) at the 5th percentile, and head circumference (39 cm) at the 25th percentile [2].

Dysmorphic features included upward-slanting eyes, a broad forehead, low-set ears, hypertelorism, a flat nasal bridge, and micrognathia. She exhibited hypotonia, head lag, and a frog-leg position. No antenatal scans were available for the affected children.

**Fig. 1:**
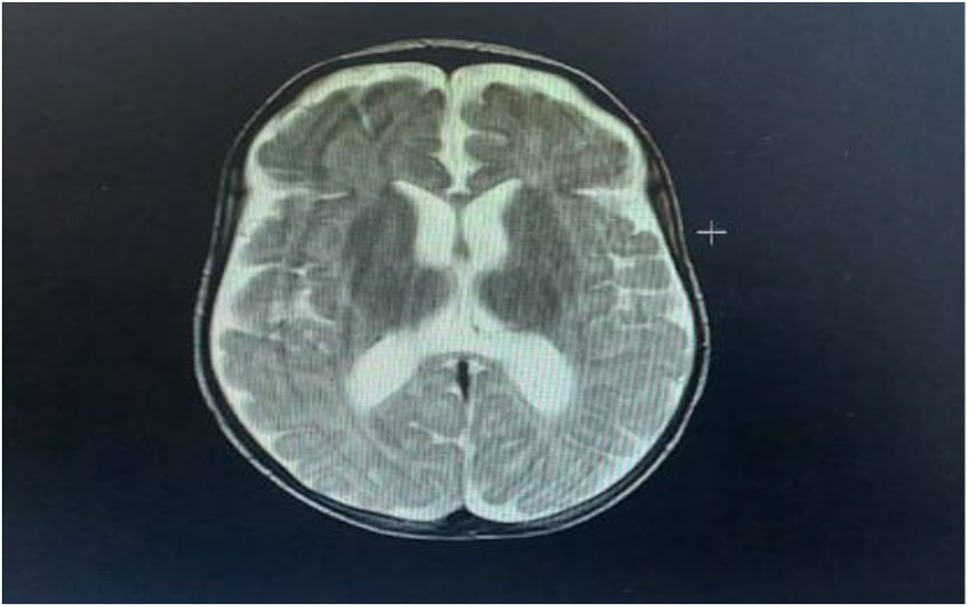
MRI brain of the patient (Note prominent cortical sulci and scant deep periventricular white matter)

A palpable abdominal enlargement was noted in the right hypochondrial region. An abdominal US confirmed hepatomegaly, with liver span measuring 15 cm.

The patient also exhibited progressive skin darkening over time (face, oral cavity mucous membrane and the tongue), hyperkalemia, with a serum potassium level of 6.5 mmol/L, elevated adrenocorticotropic hormone (ACTH) levels (44.4 pg/mL) and low cortisol levels (5 µg/dL), confirming adrenal insufficiency.

Investigations showed- Karyotyping: 46, XX; liver enzymes: elevated (SGOT 144 U/L and SGPT 267 U/L) (Normal values: 20 U/L and 25 U/L, respectively); extended metabolic screen: elevated very long-chain fatty acids (C24 at 0.1 µmol/L and C26 at 0.16 µmol/L) (Normal values: 0-0.03 µmol/L and 0.02 µmol/L respectively).

Genomic DNA was extracted from peripheral blood leukocytes and subjected to whole-exome sequencing analysis. This analysis identified a homozygous pathogenic variant in the *HSD17B4* gene, specifically the NM_001199291.1: c.1444A>T variant, resulting in an amino acid substitution from asparagine (Asn) to tyrosine (Tyr) at position 482. Based on the Human Gene Mutation Database (HGMD) Professional 2022.1 and the 2015 American College of Medical Genetics and Genomics (ACMG) guidelines, this variant is classified as pathogenic (reported mutation) [3, 4].

Fundus examination was free and hearing assessment was normal. Magnetic resonance imaging (MRI) of the brain showed prominent cortical sulci, widened sylvian fissures, and enlarged subarachnoid cerebrospinal fluid (CSF) spaces. Additionally, scant deep periventricular white matter was noted (Fig. 1).

Electroencephalography (EEG) showed centrotemporal epileptiform discharges and focal epileptiform discharges.

At the age of 1.5 mo, medium-chain triglyceride (MCT) formula along with L-carnitine and cofactors were initiated. This treatment led to significant improvement, with hepatomegaly reducing to 10 cm and normalization of liver enzyme levels [(SGOT 144 U/L→12 U/L), (SGPT 267 U/L→15 U/L)].

Regression of liver size was noticed within two weeks of formula initiation during the routine daily examination; also reduction of liver enzymes with normalization took place in five to six months.

The parents were thoroughly counseled regarding the risk of recurrence and are currently preparing for preimplantation genetic diagnosis (PGD).

## Discussion

Early neonatal onset, consanguinity, and a sibling’s death from uncontrolled seizures strongly suggest an inborn error of metabolism. Key features like hypotonia, hepatomegaly, dysmorphic traits, and elevated VLCFAs pointed to a peroxisomal disorder. The patient exhibited distinctive facial features, neurodevelopmental delay, seizures, progressive hepatomegaly, and adrenal failure, suggesting Zellweger spectrum disorder, though renal cysts were absent, ruling out Zellweger syndrome.

Down syndrome and storage diseases were excluded *via* karyotyping and clinical findings. Molecular diagnosis confirmed diagnosis of D-bifunctional protein deficiency.

Currently, there is no curative treatment for many Zellweger spectrum disorders, so treatment is generally limited to symptomatic and supportive care [5].

Mitochondrial β-oxidation of fatty acids disturbances can be managed with a diet enriched in MCTs [6].

## Conclusions

D-bifunctional protein deficiency is a rare and serious condition, with diagnosis typically confirmed through *HSD17B4* gene sequencing. This case highlights the importance of early recognition and diagnosis of DBP deficiency, especially in neonates with intractable seizures and hypotonia. The use of MCT-containing formulas may offer a promising treatment avenue, as demonstrated by the patient’s clinical improvement. The authors recommend starting this formula once diagnosis is established as Monogen (medium chain TG containing formula) is an approved formula.
