# Correction to: Neonatal Onset Seizures and Hypotonia Due to D-Bifunctional Protein Deficiency

**DOI:** 10.1007/s12098-025-05616-5

**Published:** 2025-06-08

**Authors:** Sohier Yahia, Dina Ghozzy, Yahya Wahba, Zahraa Abdelmoneim

**Affiliations:** Department of Pediatrics, Faculty of Medicine, Mansoura University, Mansoura, Egypt

**Correction to: Indian Journal of Pediatrics**

10.1007/s12098-025-05582-y

An error has been identified in the original publication of the article. The author list included a duplicate entry for ‘Sohier Yahia’.

The original article has been corrected.

